# Clinical outcome of anterior cruciate ligament reconstruction with modified transtibial and anteromedial portal

**DOI:** 10.1007/s12306-021-00727-6

**Published:** 2021-08-13

**Authors:** S. Vijayan, H. Kyalakond, M. S. Kulkarni, M. N. Aroor, S. Shetty, V. Bhat, S. K. Rao

**Affiliations:** 1 Department of Orthopedics, Kasturba Medical College, Manipal, Manipal Academy of Higher Education, Manipal, Udupi, Karnataka 576104 India; 2 Department of Orthopedics, BVVS S Nijalingappa Medical College and HSK Hospital and Research Centre, Navanagar, Bagalkot, Karnataka 587103 India; 3 Department of Physiotherapy, Manipal College of Health Professions, Manipal Academy of Higher Education, Manipal, Karnataka 576104 India

**Keywords:** Transtibial technique, Anteromedial portal, Transportal technique, ACL reconstruction, Femoral tunnel, Transfixation

## Abstract

Arthroscopic ACL reconstruction is the current standard care of treatment for anterior cruciate ligament (ACL) injuries. Modified transtibial (mTT) and anteromedial portal (AMP) techniques aim at the anatomical placement of femoral tunnel. Controversy existed in the literature with regard to the outcome of these techniques. Hence, we designed a retrospective comparative study to analyse the clinical and functional outcomes of mTT and AMP techniques. We hypothesized that there would be no difference between the clinical and functional outcomes in mTT and AMP techniques. This retrospective observational study was conducted in consecutive patients who underwent arthroscopic ACL reconstruction using semitendinosus-gracilis (STG) quadrupled graft in our tertiary care centre with a minimum follow-up of two years. Out of 69 patients, 37 had undergone ACL reconstruction by mTT technique and remaining by AMP technique. All the patients were assessed clinically by anterior drawer, Lachman’s, pivot shift and single-legged hop test. Lysholm Knee Scoring Scale and International Knee Documentation Committee (IKDC) subjective knee evaluation score were used for the functional status. Knee instability was assessed objectively by KT-1000 arthrometer. There was no statistically significant difference in baseline demographic characteristics between mTT and AMP groups. At the end of 2 years, no statistically significant difference was noted in the anterior drawer and Lachman’s test. Though not significant, IKDC scores and Lysholm’s scores showed a better outcome in the AMP group when compared to the mTT group. AMP group showed significantly better outcome with KT-1000 arthrometer. Based on the results obtained, we presume that overall both mTT and AMP have similar functional outcome. However, as AMP technique offers significantly improved subjective rotational stability on pivot shift test, better hop limb symmetry index and KT 1000 readings compared to mTT, we suggest AMP over mTT.

## Introduction

Anterior cruciate ligament (ACL) injuries are one of the most common ligament injuries occurring in young and active individuals and lead to substantial economic burden [1, 2]. ACL being an intra-articular and extra-synovial structure mainly offers anteroposterior stability apart from rotational and coronal plane stability. Untreated complete ACL tear alters the tibiofemoral kinematics and leads to functional impairment, secondary meniscal injury, chondral damage and development of early arthritis [3–6]. The peculiar anatomy of ACL, extra-synovial location, lower vascularity, lack of clot formation, retraction of torn ends by synovial myofibroblasts, poor cellular response of ligament fibroblasts in the form of lower proliferation, migration and responsiveness to growth factors and pull of quadriceps muscle impede spontaneous healing of a completely torn ACL [7]. Hence, arthroscopic ACL reconstruction, which is considered as the current standard care of treatment, becomes the viable option to restore the functional status and prevent secondary injuries of the knee [8]. The surgical outcome depends on the graft selection, thickness and length of the graft, tunnel positions in the tibia and femur, graft fixation methods in the tunnel and rehabilitation [9–11].

Anatomical positioning of the graft at the tibial and femoral footprint has been shown to have better kinematics [12]. For the creation of the femoral tunnel, five methods have been described, namely transtibial (TT), modified transtibial (mTT), anteromedial or transportal technique (AMP), outside-in (OI) technique and outside-in retrograde drilling (RD) technique [13, 14]. Since 1980, the transtibial technique was the widely used technique world over [15]. One of the problems noted with TT technique was the position of the femoral tunnel, which places the graft high in the intercondylar notch in a non-anatomical position rather than at the native ACL footprint [16]. Many authors have suggested various modifications in TT technique in an attempt to create a more anatomical femoral tunnel position termed as modified TT (mTT) technique [17–19]. Compared to TT, mTT and AMP techniques lead to more anatomical placement of the ACL graft. Various cadaveric, kinematic, biomechanical and radiological studies have assessed the difference between TT and more anatomical mTT and AMP techniques with regard to the graft placement [12, 14, 20, 21]. Many studies have given conflicting verdict about the clinical and functional outcome of both these techniques [11, 22–27]. As mTT and AMP technique aims at anatomical placement of the femoral tunnel albeit with different methods, we designed a retrospective comparative study to analyse the clinical and functional outcomes of ACL reconstruction using mTT and AMP techniques. We hypothesized that there would be no difference between the clinical and functional outcomes in mTT and AMP techniques of ACL reconstruction.

## Materials and methods

This retrospective observational study was conducted in the year 2017 using the data of patients who were operated in 2014 and 2015. Consecutive patients who underwent arthroscopic ACL reconstruction using semitendinosus-gracilis (STG) quadrupled graft in our tertiary care centre with a minimum follow-up of two years were included. Skeletally immature patients, revision ACL surgery, associated tibial plateau/femoral condyle fracture, associated intra-articular avulsion fractures of the tibial spine, posterior cruciate ligament (PCL) injuries, collateral ligament injuries, prior collateral ligament surgeries and bilateral ACL deficiencies were excluded. Data regarding ACL deficiency, which was evaluated by clinical tests and confirmed by MRI, were retrieved from the hospital medical records and PACS (picture archiving and communication system). Their surgical details were collected from the operation notes. Written informed consent was obtained from the patients. Institutional ethics committee (IEC) approval was obtained for the study. They were categorized into two groups based on the femoral tunnel drilling method, as modified transtibial (mTT) or anteromedial portal (AMP) technique, which was decided at the discretion of the operating surgeon.

### Surgical technique

All the patients were examined under anaesthesia. Objective testing was done using KT-1000, and the results were noted down. Diagnostic arthroscopy followed by meniscal balancing was done in the cases deemed necessary. All the cases were operated by a single senior surgeon. STG graft from the ipsilateral side was harvested, prepared, quadrupled and sized.

ACL remnant was debrided retaining the tibial stump. As described by Morgan et al., the tibial tunnel was drilled centring over tibial footprint using the tibial zig placed at an angle of 50° in the sagittal plane and 20° in the coronal plane with the knee flexed at 90° [28].

**Fig. 1:**
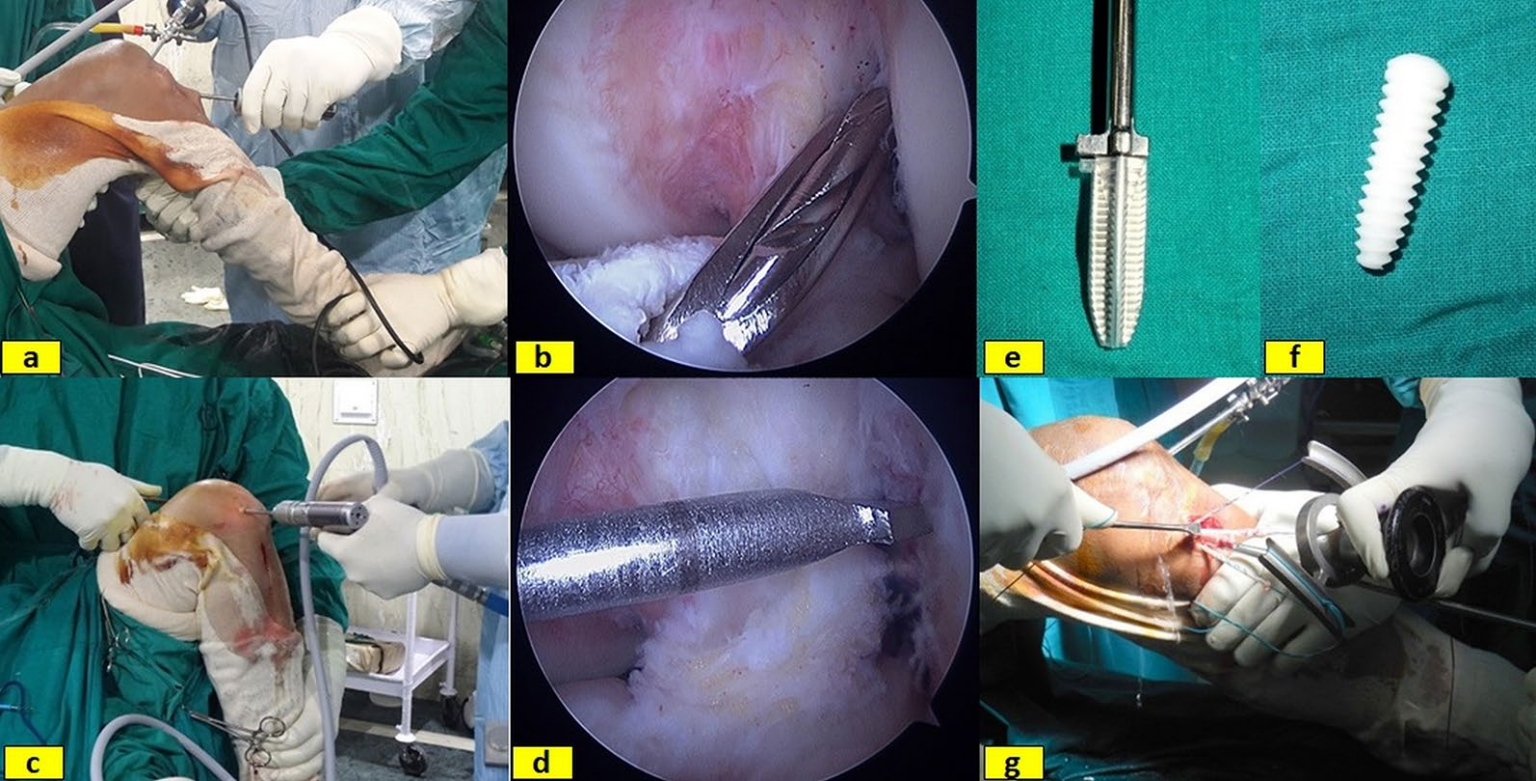
Photograph depicting external (**a**) and internal (**b**) images of femoral tunnel creation using modified transtibial (mTT) technique. External (**c**) and internal (**d**) images of femoral tunnel creation by anteromedial portal (AMP) technique. Tibial sheath (**e**), interference screw (**f**) and graft fixation in the tibial tunnel with the help of the graft tensioner (**g**)

The femoral tunnel preparation in the mTT group was made as described by Lee et al. [29]. With the knee in 90° flexion, more anatomical femoral tunnel was created by applying anterior drawer force, varus stress, external rotation of the tibia and external rotation of the femoral offset guide and directing the jig towards the anatomic centre of the ACL footprint. A guidewire was passed through the jig, and inner-to-outer cortical drilling was done with a 4-mm drill bit (Fig. 1a, b). The entire length of the femoral tunnel was measured, and femoral tunnel drilled to a depth of 35 mm corresponding to the diameter of the graft.

In the AMP group, the midpoint of the femoral footprint was identified and the calibrated guidewire was passed from that point with the knee in maximum flexion (Fig. 1c, d). The tunnel was drilled to a depth of 35 mm using the drill bit corresponding to the diameter of the graft. The length of the tunnel was marked on the graft to confirm correct insertion length. The STG graft was transfixed in the femoral tunnel using two Depuy Mitek rigidfix® femoral ST cross pins in both techniques. With the knee at 30° of flexion and a posterior drawer force applied to the proximal tibia, the graft was anchored in the tibial tunnel using Depuy bio-absorbable interference screw with sheath maintaining a tension of 15 lbs with the help of a tensioner (Fig. 1e–g). Post-operatively compression dressing and knee immobilizer were applied. The post-operative protocol was same for both the techniques (Table 1).

**Table 1:** Post-operative rehabilitation protocol for both the groups

1st week: Isometric and short arc quadriceps (300 short of full extension), auto-assisted knee flexion
2nd week and 3rd week: Resisted quadriceps exercises, sitting knee flexion, static quadriceps at 80° of flexion, prone knee bends, single-leg bridges
4th week: Full weight-bearing walking, stationary cycling with both legs 30° short of full extension, backward walking, wall sits 0–45°
8th week: single 2/3 knee bends, 6-inch step-ups, resisted quadriceps full ROM, stationary cycling with resistance
12th week: Wobble board, mini trampoline, jogging if quadriceps is at least 65% normal
16th week: Cariocas, jumping rope, resisted quadriceps throughout ROM^a^
6 months: Sports-specific activities if the knee is stable
8 months: Pivoting sports
9 months: Unrestricted sports

^a^*ROM* range of movement

At the final follow-up, two years after the index procedure, all patients were assessed clinically by a trained orthopaedic resident who was not involved in the initial surgical management of these patients. Anterior drawer, Lachman’s, pivot shift and single-legged hop symmetry index were tested to assess the stability of the operated knee. Lysholm Knee Scoring Scale and International Knee Documentation Committee (IKDC) subjective knee evaluation score were used to document the functional status [1]. Knee instability was assessed objectively by KT-1000 arthrometer. KT-1000 arthrometer values at 20 lbs (89 N) were recorded, and side-to-side difference was calculated. (Values were expressed as the difference between the operated and healthy knees). Hop limb symmetry index (LSI) as described by Noyes et al. [30] was expressed as the percentage of the longest ‘involved limb’ hop distance divided by the longest ‘uninvolved limb’ hop distance.

### Statistical analysis

Descriptive statistics were used to summarize the characteristics of both groups. Categorical variables of the clinical tests (anterior drawer, Lachman and pivot shift tests), Lysholm knee score and KT-1000 arthrometer measurements were compared using Fisher’s exact test. Independent-samples test was employed to compare both groups for single-legged hop test, IKDC scores and KT-1000 readings. SPSS Statistics, IBM Corp®. version 20.0 was used to analyse the data. In all analyses, the *p* value of < 0.05 indicated a statistically significant difference. Post hoc power analysis was done using OpenEpi version 3.

## Results

Of the 81 consecutive patients, only 76 fulfilled the inclusion criteria. (One case each of revision ACL reconstruction, tibial plateau fracture, contralateral ACL reconstruction and two cases of MCL repair were excluded.) Seven cases were not having follow-up of 2 years, and only the remaining 69 cases were considered for the study.

The majority were males (62/69), having the involvement of the right knee (41/69). Mean age of the patients was 30.22 (± 9.34) yrs. Road traffic accident (RTA) was the most common mode of injury (32/69) followed by sports injuries and fall. Out of 69 patients, 42 had associated meniscal involvement. Out of 42 cases, 13 had involvement of both the menisci. Out of 69 patients, 37 had undergone ACL reconstruction by mTT technique and remaining by AMP technique (Table 2).

**Table 2:** Demographic distribution of patients

Variables	All patients (**± **SD)
No. of patients	69
Male/female	62/7
Age in years	30.22 (± 9.34)
Affected side	
Right/left	41/28
Mode of injury	
RTA^a^	32 (46.4%)
Fall	17 (24.6%)
Sports injury	20 (29%)
Meniscus injury	42/69
Medial meniscus	20
Lateral meniscus	09
Both Menisci	13
Duration in months	12.77 (± 15.88)
Surgical technique	
Modified transtibial	37
Anteromedial portal	32

^a^*RTA* road traffic accident

**Fig. 2:**
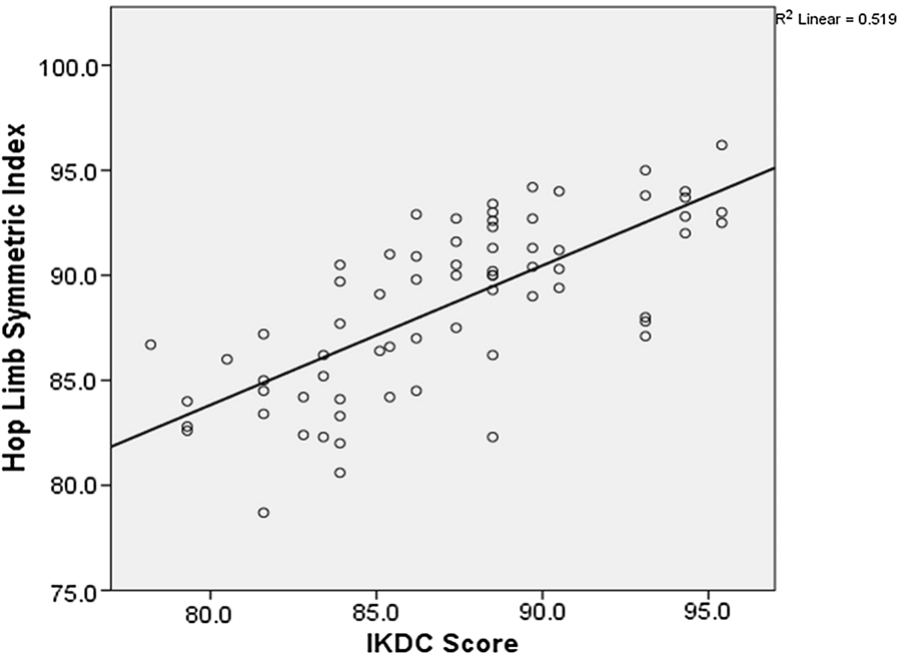
Scatter plot showing the correlation between hop limb symmetry index and IKDC score

There was no statistically significant difference between mTT and AMP groups with respect to the age, gender, duration since the injury, affected side, involvement of the meniscus, Lachman test, anterior drawers test and KT 1000 values (Table 3). At the end of 2 years, no statistically significant difference was noted in the anterior drawer and Lachman’s test among the two groups. IKDC scores were better in the AMP group, but the difference was not statistically significant. It was also observed that the average IKDC score in patients with isolated ACL injury (88.57) was better than in those associated with meniscal injuries (86.28). Lysholm’s scores showed a better outcome in the AMP group with 75.4% patients having excellent scores compared to mTT group in which only 62.2% of patients had excellent scores. However, this was not statistically significant. In our series, we had no grade 2 or 3 pivot shift positive cases. Grade 1 positive pivot shift was observed in 11 patients in the mTT group and 3 patients in the AMP group, and this difference was noted to be statistically significant. Average hop limb symmetry index (LSI) was 87.26 for the mTT group and 90.13 for the AMP group, and the difference was statistically significant, thereby inferring that AMP technique offers better dynamic knee stability as compared to mTT technique. Also, KT-1000 arthrometer values in the AMP group showed significantly better results compared to mTT group, thereby suggesting that AMP technique offers better anteroposterior and rotational stability in comparison with the mTT technique (Table 4). We also evaluated the correlation among the measuring tools. Single-hop limb symmetry index, IKDC score and KT-1000 measurements all were correlating significantly and in agreement with each other (Fig. 2) and (Table 5).

**Table 3:** Comparison of demographic and preoperative evaluation findings between the two groups

	Total	mTT^a^	AMP^b^	*p* value
Gender				0.696
Male	62	34 (91.9%)	28 (87.5%)	
Female	7	3 (8.1%)	4 (12.5%)	
Side affected				1.000
Right	41	22 (59.5%)	19 (59.4%)	
Left	28	15 (40.5%)	13 (40.6%)	
Age in years	30.22 (± 9.34)	29.03 (± 9.18)	31.59 (± 9.46)	0.258
Duration in months	12.77 (± 15.88)	13.02 (± 14.51)	12.49 (± 17.56)	0.892
Meniscus status				
Medial meniscus				0.148
Healthy	36	16 (43.4%)	20 (62.5%)	
Torn	33	21 (56.8%)	12 (37.5%)	
Lat Meniscus				1.000
Healthy	47	25 (67.6%)	22 (68.8%)	
Torn	22	12 (32.4%)	10 (31.2%)	
*Preoperative anterior drawers test*	*56*	*30*	*26*	*0.531*
*Grade 0*	*0*	*0*	*0*	
*Grade 1*	*1*	*0*	*1 (3.8%)*	
*Grade 2*	*32*	*18 (60%)*	*14 (53.8%)*	
*Grade 3*	*23*	*12 (40%)*	*11 (42.3%)*	
*Preoperative Lachman test*	*56*	*30*	*26*	*0.601*
*Grade 0*	*0*	*0*	*0*	
*Grade 1*	*0*	*0*	*0*	
*Grade 2*	*26*	*15 (50%)*	*11 (42.3%)*	
*Grade 3*	*30*	*15 (50%)*	*15 (57.7%)*	
KT-1000 (Preoperative) (mm)	7.39 (± 1.82)	7.54 (± 1.86)	7.22 (± 1.79)	0.46

*p < 0.05 is considered as statistically significant

^a^*mTT* modified transtibial technique

^b^*AMP* anteromedial portal technique

**Table 4:** Comparison of post-operative clinical evaluation and functional outcome among the two groups

	All the patients	mTT^a^	AMP^b^	*p* value
Post-operative anterior drawers test				0.77
Grade 0	42	21 (56.8%)	21 (65.6%)	
Grade 1	24	14 (37.8%)	10 (31.3%)	
Grade 2	3	2 (5.4%)	1(3.1%)	
Grade 3	0	0	0	
Post-operative Lachman test				0.59
Grade 0	34	16 (43.2%)	18 (56.3%)	
Grade 1	29	17 (45.9%)	12 (37.5%)	
Grade 2	6	4 (10.8%)	2 (6.3%)	
Grade 3	0	0	0	
Post-operative pivot shift test				0.04*
Grade 0	55	26 (70.3%)	29 (90.6%)	
Grade 1	14	11 (29.7%)	3 (9.4%)	
Grade 2	0	0	0	
Grade 3	0	0	0	
KT-1000 (2-year follow-up) (mm)	1.78 (± 0.92)	2.14 (± 0.88)	1.38 (± 0.79)	0.000*
Post-operative KT-1000 values at 20 lbs (89 N)				0.007*
< 3-mm side-to-side difference	54	24 (64.9%)	30 (93.8%)	
3–5-mm side-to-side difference	15	13 (35.1%)	02 (6.3%)	
Hop limb symmetry index	88.59 (± 4.06)	87.26 (± 4.04)	90.12 (± 3.56)	0.003*
IKDC score	87.17 (± 4.41)	86.34 (± 3.97)	88.13 (± 4.74)	0.092
Post-operative Lysholm’s score				0.56
Excellent	47	23 (62.2%)	24 75.0%)	
Good	19	12 (32.4%)	07 (21.9%)	
Fair	3	2 (5.4%)	1 (3.1%)	
Poor	0	0	0	

*p < 0.05 Student’s *t* test

^a^*mTT* modified transtibial technique

^b^*AMP* anteromedial portal technique

**Table 5:** Correlation among the functional assessment tools

Correlation	Pearson correlation	*p*-value	*R*^2a^
Hop limb symmetry index Vs IKDC score	0.721	0.000^*^	0.519
Hop limb symmetry index Vs KT-1000	− 0.686	0.000^*^	0.470
KT-1000 Vs IKDC score	− 0.668	0.000^*^	0.447

*p < 0.05

^a^*R*^2^: correlation coefficient

Our retrospective study had 37 patients in mTT and 32 patients in the AMP group. Post hoc power analysis showed that our study was adequately powered for hop limb symmetry index (87.82%) and KT 1000 arthrometer readings (96.56%) with an alpha error of 5% by the normal approximation method.

## Discussion

Many cadaveric, biomechanical, kinematic and radiological studies have revealed the advantages of anatomical positioning of the ACL graft [12, 31, 32]. It has been observed that the TT technique places the graft in a non-anatomical position in the intercondylar region [10, 16, 23, 31]. Due to the tibial tunnel constraints, femoral tunnel will be placed more superior and anterior in the femoral condyle by TT technique [33, 34]. AMP technique facilitates femoral tunnel drilling in the centre of the footprint and hence anatomical positioning of the graft [12, 16, 23, 31, 35]. Vertical placement of the graft in TT technique gives adequate anteroposterior stability for the knee, but limited rotational stability [11, 18, 24]. However, improved anteroposterior, as well as rotational stability, can be achieved by slight horizontal placement of the graft as in the AMP technique [32, 36–38]. This finding is corroborated by Schairer et al. [21] in their in vivo study of reconstructed ACL by AMP and TT technique using MRI. Shorter femoral tunnel, higher risk of posterior wall blow out, the requirement of high flexion of the knee and scuffing of the medial femoral condyle by the drill are few of the criticism against the AMP technique [22, 34].

The posterolateral bundle of ACL along with anterolateral ligament complex (iliotibial band with its deep and capsulo-osseous layer, Kaplan fibres, lateral capsule, and mid-third capsular ligament), posterior horn of lateral and medial meniscus and bony morphology of distal femur provide rotatory stability to the knee joint in extension [39–41]. The anatomic double-bundle ACL reconstruction technique by virtue of recreating posterolateral bundle shows better rotational stability than single-bundle technique [39–42]. However, evidence for clinical and biomechanical benefits of double-bundle ACL reconstruction is still debated to date [41]. Sonnery-Cottet et al. [42] described that performing an additional extra-articular lateral reconstruction procedure along with standard single-bundle ACL reconstruction is more effective than isolated single- or anatomic double-bundle reconstruction and prevented the displacement of the lateral tibial compartment. But, some studies have failed to demonstrate outcomes in favour of the lateral tenodesis too [41, 42].

Many authors have described various modifications in TT technique to achieve more anatomical position of the femoral tunnel. Medializing and taking more proximal entry point for the tibial tunnel is one such technique. Injury to the MCL, widened tibial aperture and shorter tibial tunnel are the risks involved in this method [43]. Another described method is to use a smaller femoral drill and do incremental drilling by readjusting the guidewire so that it has some play in the larger tibial tunnel and thereby try to achieve better anatomical position [17, 44]. Chung et al. [45] suggested a larger femoral offset guide to achieve anatomical femoral tunnel positioning. Lee et al. [29] described manoeuvre, which can be used to achieve more anatomical positioning of the femoral tunnel. They suggested applying anterior drawer and varus force along with an external rotation of the tibia and external rotation of the femoral offset guide to position the femoral tunnel more posteriorly and horizontally than regular TT technique.

In our study, we found no statistical difference in IKDC score, Lysholm’s score and anterior drawers test results between mTT and AMP groups. Our results are similar to the conclusion drawn by Youm et al. and Han et al. [46, 47]. Though in the AMP group the scores were better, it was not statistically significant. Closer scrutinization of the results reveals that the AMP group showed significantly better pivot shift, single-hop limb index scores and KT-1000 results. This is in contrast to the finding of Lee et al. [19] who found no difference between the two groups. We had no cases having KT-1000 laxity > 5 mm, which is similar to the observation made by Youm et al. [46]. But we noted that mTT had significantly more cases which had 3- to 5-mm side-to-side laxity (mTT 13/37 vs AMP 2/32; *p* < 0.005).

Though mTT aimed at producing anatomical femoral tunnel comparable to AMP technique, the femoral tunnels that are produced by mTT are less oblique compared to AMP technique [43]. Increased obliquity of the graft placement by AMP technique improves the rotational stability, which has been proved by biomechanical studies as well [37]. This improved rotational stability due to increased obliquity of the graft placement in AMP technique can explain the difference seen in our results.

We used the ST cross pins for transfixing the graft in the femoral tunnel, which allows for maximum contact between the graft and the host bone avoiding any dead space in the tunnel. And it also addresses issues with posterior wall blow out seen mostly with AMP technique. Hyper flexing the knee to 110° while drilling the femoral tunnel will result in creation of a tunnel of sufficient length by AMP technique [48]. Other criticism on AMP is about damaging the medial femoral condyle cartilage while drilling the femoral tunnel. This can be avoided by careful technique and making low AM portal or 2 AM portals and start drilling only once the drill bit contacts the femoral footprint [26].

In our study, we did not have any major complications. Postoperatively, none of our patients had grade 3 instability to suggest a re-rupture of the graft. In two patients, we had to remove the tibial interference screw due to its prominence. But since the patients became symptomatic only after more than 2 years, it did not change the outcome.

Strengths of this study are that it is a single-centre study, all cases operated by an experienced single surgeon and all patients were managed by a standard protocol. There was no significant demographic difference among the group with the acceptable number of loss of follow-up of the cases (90.8% completed the follow-up at 2 yrs). Both the groups were identical and comparable except for the technique for the femoral tunnel drilling**.** Post hoc power analysis revealed that our study was adequately powered. Our study has few limitations. Ours is a retrospective observational study. We did not evaluate for the position of the graft and tunnels radiologically in the post-op period, which would have confirmed the difference in the graft positioning among these two techniques. We found it an unnecessary exercise as there are many studies in the literature, which already have proven the point [47]. Ours is a short-term study, which is not expected to reveal the prevention of further injuries to the meniscus and development of osteoarthritis changes. A well-constructed double-blinded randomized controlled trial with an adequate number of participants comparing mTT and AMP techniques with sufficient long-term follow-up can address these issues.

Based on the results obtained, we believe that overall both mTT and AMP have similar functional outcome. We do acknowledge that our study has its own inherent limitations due to the retrospective non-randomized design and short-term follow-up. But, as AMP technique offers improved subjective rotational stability on pivot shift test, better hop limb symmetry index and KT 1000 readings at 2 years, we suggest AMP over mTT.
